# Material-assisted metamaterial: a new dimension to create functional metamaterial

**DOI:** 10.1038/srep42076

**Published:** 2017-02-06

**Authors:** Wei-Yi Tsai, Chih-Ming Wang, Ching-Fu Chen, Pin Chieh Wu, Yi-Hao Chen, Ting-Yu Chen, Pei Ru Wu, Jia-Wern Chen, Din Ping Tsai

**Affiliations:** 1 Department of Physics, National Taiwan University, Taipei 10617, Taiwan; 2 Department of Opto-electronic Engineering, National Dong Hwa University, Hualien 97401, Taiwan; 3 Research Center for Applied Sciences, Academia Sinica, Taipei 11529, Taiwan; 4 College of Engineering, Chang Gung University, Taoyuan 33302, Taiwan

## Abstract

A high Q-value reflective type metasurface consisting of 1D Au nanorods, a SiO_2_ spacer and a Au back reflector is demonstrated. It is shown that the sideband of the resonant mode can be suppressed as the resonant wavelength close to the phonon absorption of SiO_2_. By combining both designed structured resonance and inherent property of the based materials, a low angle-dependent metasurface with a Q-value of 40 has been demonstrated. The proposed structure will be useful for high sensitivity sensing and narrow band thermal emitter.

Metamaterials, with functional electromagnetic behaviors on demand, are typically engineered by arranging a set of arbitrary designed structures in a regular array throughout a region of space^1^,^2^,^3^. Recently, the research activities extended toward to planar metamaterials also known as metasurfaces, i.e. with arbitrarily designed structures at a surface or interface. Metasurfaces have the advantage of taking up less physical space and simpler geometry than three-dimensional metamaterial structures. Consequently, the metasurfaces suffer less challenge of fabrications. Metasurfaces have been demonstrated in various potential applications, such as miniaturized cavity resonators^4^, plasmonic wave-guiding structure^5^,^6^, perfect absorbers^7^,^8^,^9^, biomedical devices^10^, terahertz switches^11^,^12^,^13^, fluid-tunable frequency-agile materials^14^, high efficiency hologram^15^,^16^,^17^, phase change materials^18^,^19^, as well as spectrally selective thermal emitters^20^,^21^.

Very recently, Giessen *et al*. propose chemical method to growth Au plate with atomic-level roughness^22^,^23^. The propagation loss and material loss of metamaterials as well as plasmonic circuits can be significantly reduced. Usually, people say that the unique electromagnetic response of a metamaterial is from the arbitrarily designed structure but not from the inherent property of based materials. However, apparently, materials do affect the performance of metamaterials. Consequently, utilizing material inherent property can be an alternative to modify the resonance behavior of metamaterials.

Scientists have investigated a variety of metamaterial and metasurface structures in order to obtain custom tailored responses for spectral filtering and sensing applications^24^,^25^,^26^,^27^. Achieving narrow resonance by using metamaterials as well as metasurfaces is useful for both fundamental research and practical applications. Especially, the widely investigated split ring resonator (SRR) based designs show a strong angular dependence^28^, as the transmission properties of these structures rely in part on a coupling with a surface electromagnetic wave. Moreover, this kind of structure is usually with low quality factors due to the symmetry and inherent loss of utilized material^29^,^30^. Here, the Q-value refers to the ratio between the center frequency and full width at half-maximum (FWHM) bandwidth of a resonance. Usually, the Q-value of the symmetrical SRR operated in THz range is generally less than 10. Symmetry breaking SRRs have been proposed to improve the Q-value of planar SRR structures^31^. In order to furthering achieve a metamaterial with high Q-value, dielectric metasurface has been demonstrated^32^.

In this paper, we demonstrate a magnetically resonant metasurface with a high Q-value combining both designed structured resonance and inherent property of the based materials. By using this unique design rule, a high Q-value metasurface with low angle-dependence has been demonstrated. The proposed structure will be useful for high sensitivity sensing and narrow band thermal emitter.

## Results

### Material-assisted metamaterial

**Figure 1:**
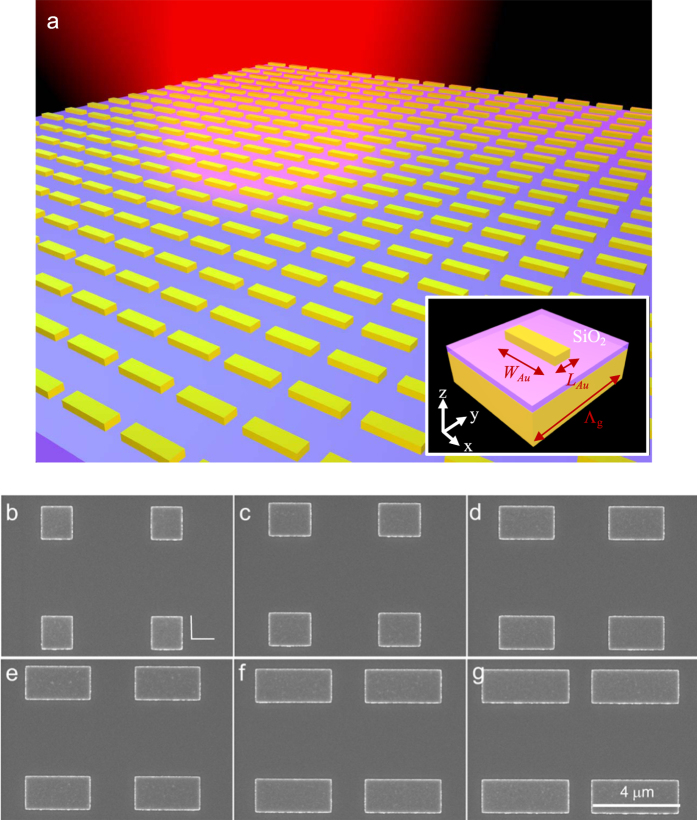
Side-view diagram of the investigated 1D metasurface. The width of Au wire is denoted by *W*_*Au*_. The incident angle is θ_in_. (**b**–**g**) Are the SEM images of Au nanorod, and the *W*_*Au*_ are 1.5 μm, 2 μm, 2.5 μm, 3 μm, 3.5 μm, and 4 μm, respectively. The scale bar of the SEM pictures are all fixed to be 4 μm.

The schematic of the investigated metasurface is shown in Fig. 1a. The structure consists of a SiO_2_ spacer layer sandwiched by Au nanorod array and Au thin film. The nanorod array supporting dipole reosonances is one of the simplest metasurface structure. The thickness of SiO_2_ layer and Au rod is 50 nm and 100 nm, respectively. The width and length of the Au rod are denoted by *W*_*Au*_ and *L*_*Au*_, respectively. The periodicity of the structure is denoted by ***Λ***_*g*_. Here, the periodicity is 5 μm. The distance between the nanorods is so long that the nanarods are not coupled. Conventionally, a well-defined nano-gap between two adjacent metallic structures, for example, bow-tie and dimer structures, relies on precisely E-beam lithography. Here, the plasmonic coupling effect occurs in between the nanorod and Au film. Using the sputtering deposition technology, the gap between the two metallic structures can be precisely defined. This makes us able to investigate the property of the gap-plasmon resonance more stable. The thickness of SiO_2_ layer plays an important role for the near-field interaction between upper metallic rod and its image charges induced by the metallic mirror. It has been demonstrated that a thinner 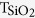 usually leads to a higher localized field enhancement^33^. Here, we simply fix the thickness to be 50 nm without optimizing. We controlled the plasmonic response of the metasurfaces by varying the width of the Au nanorod (denoted by *W*_*Au*_). The thickness of the Au nanorod is kept to be 100 nm. To investigate the dependence of the device properties on the width of the Au rod, we fabricated six samples with *W*_*Au*_ = 1.5 μm, 2.0 μm, 2.5 μm, 3.0 μm, 3.5 μm and 4 μm, while keeping the periodicity at ***Λ***_*g*_ = 5 μm and the width *W*_*Au*_ = 1.5 μm, as shown in the scanning electron microscope (SEM) pictures (Fig. 1b–g). The area of each sample is 150 μm × 150 μm.

### Optical characterization of the Material-assisted metamaterial

**Figure 2:**
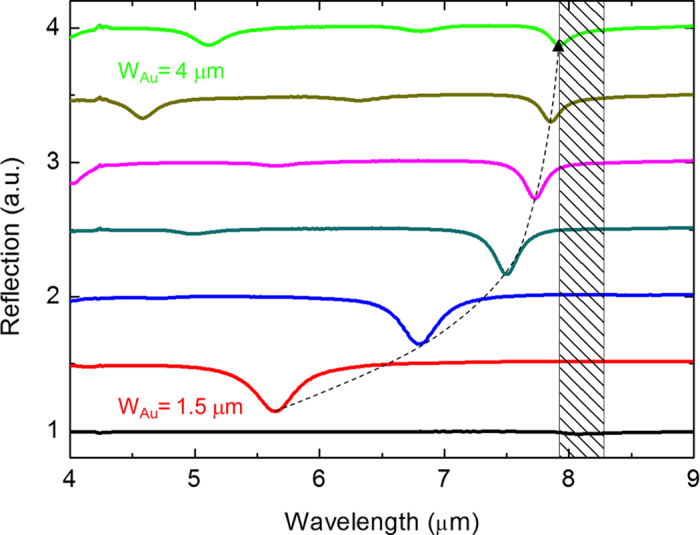
Experimental reflectance spectrum of 1D metasurfaces with a series of W_Au_. The measurement results are obtained under normal incident with x polarized. Black line is SiO_2_ with a thickness of 50 nm coated on Au film for reference. Red, orange, yellow, green and blue lines indicate the reflectance of metasurfaces with *W*_*Au*_ = 1.5 μm, 2.0 μm, 2.5 μm, 3.0 μm, 3.5 μm and 4 μm, respectively. The period is fixed to be Λ_g_ = 5 μm.

The fabricated samples are measured using micro Fourier transform infrared spectroscopy (μ-FTIR). Figure 2 shows the reflection spectra of the investigated structures under *x*-polarization, i.e. the electric field oscillating along the long axis of the nanorod. The thickness of the SiO_2_ layer is kept to be 50 nm for every sample. For reference, the black line shows in the FTIR absorption spectrum of a silicon substrate sequentially coated with 100 nm Au and 50 nm SiO_2_. It is shown a weak reflection dip at 8.1 μm which is due to the inherent absorption of SiO_2_.

The red, orange, yellow, green and blue solid lines represent the reflection spectrum for W_Au_ = 1.5 μm, 2.0 μm, 2.5 μm, 3.0 μm 3.5 μm, and 4 μm, respectively. Spectra are gradually offset with 0.4 in the y-axis for clarity. The period of the Au nanorod array is fixed to be ***Λ***_*g*_ = 5 μm. It shows a clear resonance peak at *λ* = 5.63 μm for *W*_*Au*_ = 1.5 μm. The resonance is due to the near-field interaction between the induced resonance current at upper metallic nanorod and image currents induced at the metallic mirror. A magnetic dipole resonance can be excited due to the two metallic layers. This mode is assigned to be a fundamental Fabry-Perot (FP) gap-plasmon mode inside the MIM cavity formed by the two adjacent metallic structures. At this spectral range, the absorption coefficient of SiO_2_ is ignorable. The absorption mostly comes of the Ohmic heating inducing from the gap-plasmon resonance, i.e., the magnetic dipole resonance in between the two metallic structures. Spectral scaling can be simply achieved by tuning the cavity length or the gap thickness of the MIM cavity structures. Here, by tuning the length of the Au nanorod, we are able to manipulate the gap-plasmon resonance wavelength. Increasing the cavity length shifts the gap-plasmon modes toward longer wavelengths. It is shown that the gap-plasmon resonance wavelength shifts from 5.63 μm to 7.92 μm as a *W*_*Au*_ sweep from 1.5 μm to 4 μm. Beside of the red-shifting resonance wavelength, it can be seen that the linewidth of the resonance dips significantly decreased.

### Enhanced Q-value by suppressing the bandwidth of resonance

**Figure 3:**
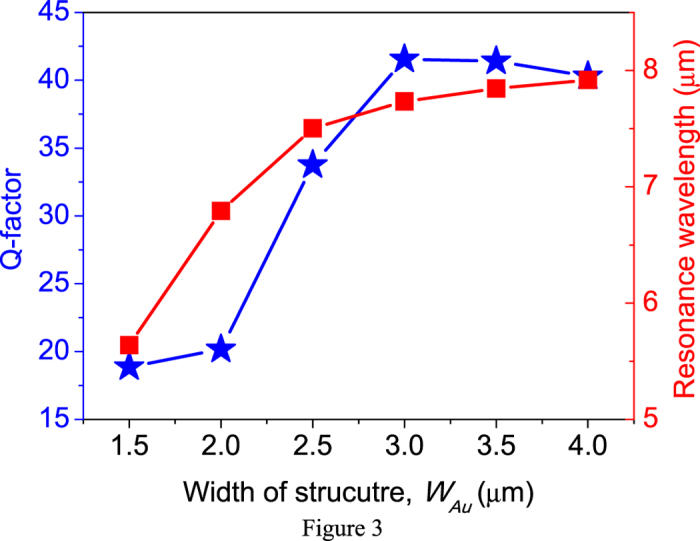
Quality factors and resonance wavelengths of the fundamental FP modes as a function of W_Au_.

The Q-values and the resonance wavelengths of the fundamental FP modes of the gap-plasmon resonances are shown in Fig. 3. As mentioned before, the resonance wavelength can be increased as an increasing *W*_*Au*_. For *W*_*Au*_ increasing from 1.5 μm to 2 μm, the resonance wavelength shifts 1.1 μm. The ratio between the wavelength shift and *W*_*Au*_ increase can be simply regarded as the effective index of the plasmonic MIM cavity for no loss dielectric layer^33^. Here, due to the plasmonic coupling effect, the effective index is 2.64 which is much higher than the refractive index of bulk SiO_2_. For *W*_*Au*_ increasing from 3.0 μm to 3.5 μm, the resonance wavelength only slightly shifts 0.12 μm. At this time, the gap-plasmon resonance is close to the inherent absorption of SiO_2_, the near-field coupling at the two metallic layers is inefficient due to the absorption. Therefore, the resonance wavelength shift is less significantly compared to that far-away from the absorption peak of SiO_2_. It also can be seen that the Q-value almost the same as *W*_*Au*_ increasing from 1.5 μm to 2 μm. The Q-value is limited by the inherent absorption of Ohmic heating as gap-plasmon resonance. As the *W*_*Au*_ is kept increasing from 1.5 μm to 4.0 μm, the Q-value dramatically increases from 18.85 to 40.29. This is because that the band tail of the gap-plasmon resonance at long wavelength regime is suppressed as it is close to the absorption wavelength of lossy SiO_2_. Similarly, as *W*_*Au*_ increasing from 3.0 μm to 3.5 μm, the Q-value maintains almost the same. Thanks to the inherent absorption of the dielectric layer, we can enhance the Q-value of metasurfaces by a factor up to 2-folds.

**Figure 4:**
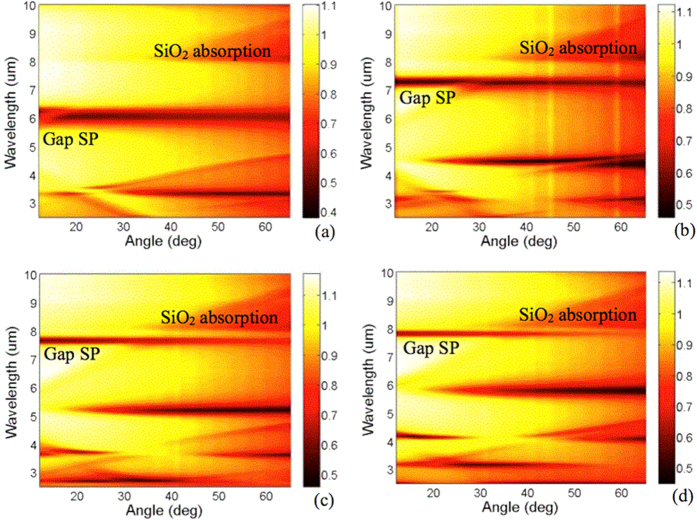
Measurement results of Angle resolved reflection spectrum of the 1D metasurfaces. (**a**–**d**) for *W*_*Au*_ = 2 μm, 2.5 μm, 3.0 μm and 3.5 mm, respectively. The colorbar represents the reflectance of the metasurfaces.

In order to observe the influence between the gap plasmon and phonon vibration in detail, the angle-resulted FTIR is applied. For simplifying the influence of input polarization, we investigate a Au wire array so that TM-polarized light, i.e. the electric field parallel to the k-vector of the periodic Au wires, can arise the magnetic resonance mode in between the uppermost Au wires and the bottom Au reflector. The width of the Au wire is identical to the Au nanorod. For *W*_*Au*_ = 2.0 μm, the gap-plasmon is far away from absorption of SiO_2_ from λ = 8.1 μm to λ = 9.6 μm, as shown in Fig. 4a. The gap-plasmon resonance present a broad resonant line shape. For *W*_*Au*_ = 2.5 μm (as shown in Fig. 4b), the gap-plasmon resonance redshift toward the absorption of SiO_2_. Although the resonance wavelength, 7.3 μm, is still away from it. One can still observe that the linewidth of the resonance is significantly reduced. For *W*_*Au*_ = 3.0 μm, the linewidth of the resonance dip is further reduced. For *W*_*Au*_ = 3.5 μm, the gap-plasmon resonance is very close to the absorption wavelength of SiO_2_. It is shown that there is a bright line between the gap plasmon and the absorption of SiO_2_. It is an evident that there is no coupling effect between the gap-plasmon and the SiO_2_ absorption. Additionally, the resonance becomes sharp and non-dispersive under oblique observation angles.

According to the ref. 34, the real parts of the complex refractive index of SiO_2_ is smaller than 1. The SiO_2_ is lossy due to the phonon absorption. Therefore, the gap-plasmon cannot be excited. Owing to this unique feature, we can further enhance the Q-value of metasurface via manipulating both the structure induced absorption and inherent absorption of the base material of metasurfaces. We believe that this is a new approach to obtain a high Q-value metasurface.

## Discussion

In summary, narrow band metasurfaces with lossy dielectric cavities have been investigated. The MIM cavities of the metasurfaces support FP-like resonance which can be manipulated via changing the width of the Au nanorods. Via manipulating the structure induced absorption in cooperation with the inherent absorption of the base material, we demonstrate that the Q-value can be significantly enhanced up to 2-folds by using a lossy dielectric cavity. The proposed unique method and proposed structure can be applied for highly sensitive sensing and narrow band thermal emitters.

## Methods

### Sample preparation

The narrow band metasurface was fabricated by standard E-beam lithography and lift-off processes. The metasurface was a sandwich structure and consisted of a Au film, a SiO_2_ spacer layer, and a Au nanorod rarray. By sputtering deposition technology, the Au film, adhesion layer, and SiO_2_ layer were sequentially deposited on a silicon substrate with a thickness of 150 nm, 3 nm, and 50 nm, respectively. The adhesion layer was Cr to improve the bonding strength between Au and SiO_2_. The sample was then coated with photoresist Poly-methyl methacrylate (PMMA) by spin coating, and baked for 3 minutes on a hotplate with temperature of 180 °C. Subsequently, the sample was patterned by using the e-beam system (Elionix ELS-7000) at acceleration voltage of 100 keV. After exposure, it was immersed into the solution of Methyl isobutyl ketone (MIBK) for development. The total thickness of Au nanorod is 100 nm composed of 3 nm sputter-deposited Au and 97 nm Au made by E-gun thermal deposition. Finally, the metasurface structure can be fabricated through the lift-off procedure.

### Simulation

A commercial finite element method software, COMSOL, is applied to simulate the optical property of the proposed metasurface structure. The optical properties, such as resonance spectrum, Q-value of the resonance and so on, are simulated and analyzed as the wavelength of the magnetic resonance approaching to the inherent loss of SiO_2_. The thickness of T_Au_ and 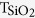 are fixed to be 100 nm and 50 nm, respectively. Here, the Q-value is defined as: 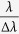, where λ is resonant frequency and Δλ is the FWHM of the resonant peak.

### Measurement

The measurement data are extracted by using Bruker VERTEX 70 Fourier-transform infrared spectrometer equipped with Bruker HYPERION 2000 infrared microscope (15× Cassegrain objective with a numerical aperture of N.A. = 0.4, near-infrared polarizer, and MCT detector). The internal iris of HYPERION microscope is used to collect the incident light to a square area of about 150 × 150 μm^2^. The reference of reflectance spectrum is gold mirror.

### Reflection spectrum for a lossless SiO_2_

For comparison, the reflection spectrum of our proposed structure with lossless SiO_2_ layer is simulated. The refractive index of SiO_2_ is described using Cauchy equation:


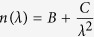


where n is the refractive index, λ is the wavelength. Here, B = 1.4580 and C = 0.00354 is taken from ref. 35. Figure S1 shows that the FWHM of the resonance peak gradually increases as the resonance wavelength gradually redshifts due to the increasing width of Au nanorod. On the contrary, the FWHM significantly becomes narrower as the loss of SiO_2_ is considered as shown in Fig. 2. This reveals that the Q-value enhancement is due to the phonon absorption of SiO_2_ at 8–12 μm.

## Additional Information

**How to cite this article**: Tsai, W.-Y. *et al*. Material-assisted metamaterial: a new dimension to create functional metamaterial. *Sci. Rep.* **7**, 42076; doi: 10.1038/srep42076 (2017).

**Publisher's note:** Springer Nature remains neutral with regard to jurisdictional claims in published maps and institutional affiliations.

## Supplementary Material

Supplementary Information
