# Supplementary material for: Material-assisted metamaterial: a new dimension to create functional metamaterial
Source: Sci Rep. 2017 Feb 6;7:42076. doi: 10.1038/srep42076 (PMC5292717; doi:10.1038/srep42076)
Supplement: Supplementary Information [file srep42076-s1.pdf]

Supporting Information

Material-assisted metamaterial: a new dimension to create functional metamaterial

Wei-Yi Tsai, Chih-Ming Wang, Ching-Fu Chen, Pin Chieh Wu, Yi-Hao Chen, Ting-Yu Chen, Pei Ru Wu, Jia-Wern Chen, and Din Ping Tsai**

Permittivity of SiO_2:

Figure S1 shows reflection spectrum and the relative permittivity of SiO_2 from ref. 23. The black line represents the reflection spectrum of the Au wire with a width of 3.5 μm under TM-polarized illumination. It can be seen that the resonance dip is suppressed for $\lambda > 8$ μm. According to the reference, the real part of permittivity becomes negative and the imaginary part becomes nonzero for $\lambda > 8$ μm. Therefore, the gap plasmon resonance supported by two Au nanostructures is suppressed.

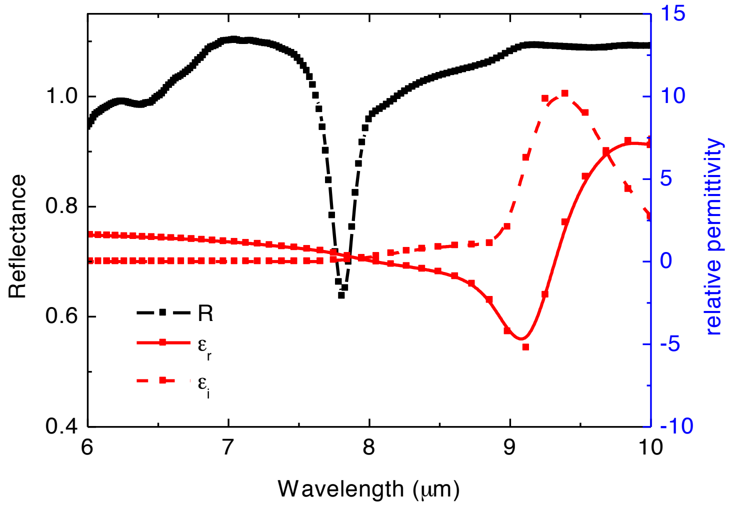

Fig. S1 Reflection spectrum of Au nano wires and the relative permittivity of SiO_2

Simulation:

In the simulation, the Multiphysics commercial software, COMSOL, is employed for the simulation of optical spectra. The exploited parameters of T_{Au}, T_{SiO2} are 100 nm and 50 nm, respectively. The resonant spectra of this structure as a function of width is shown in figure S2 (a). Once the plasmonic resonances approach to the absorption band of SiO_2, the gap-plasmon is uneffiecent and the absorption band is dominating the process of light-matter interaction; therefore, the gap-plasmon resonance and magnitude are gradually shrinked and reduced, respectively. Because the absorption band as obstacle for the plasmonic resonance which restrict the plasmonics across it. In general, the definition of quality factor is defined as: $\frac{f}{\Delta f}$. Where the f is resonant frequency and Δf is the FWHM. The gap-plasmon is narrower, the enhancement of quality factor is higher. This shrinked effect provides a novel method to suppress the resonance of gap-plasmon and enhances the performance of quality factor. The comparison of quality factor between experimental measurement and simulation results are shown figure S2 (b). It is shown that both of the simulation and measurement results show similar trend as an increasing W_T. However, the magnitude of the experimental results is much lower than the simulation one. That might be because the uniformity of the fabricated sample and the Ohm loss of Au.

The Ohm loss of Au (by thermal evaporation deposition) can be significantly reduced by using a single crystalline Au.

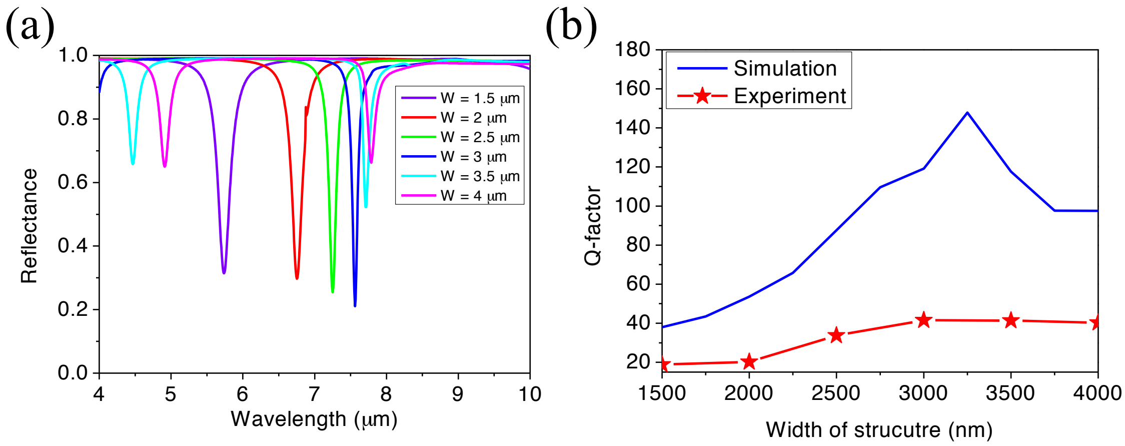

Fig. S2(a) the reflectance spectrum of Au nanowires in the simulation and (b) the comparison of Q-factor between simulation and experimental results.

Reflection spectrum for a lossless SiO2

For comparison, the reflection spectrum of our proposed structure with lossless SiO2 layer is simulated. The refractive index of SiO2 is described using Cauchy equation:

$$n(\lambda) = B + \frac{C}{\lambda^2}$$

where n is the refractive index, λ is the wavelength. Here, B = 1.4580 and C = 0.00354 is taken from ref [1]. Fig. S3, in the supplementary information, shows that the FWHM of the resonance peak gradually increases as the resonance wavelength gradually redshifts due to the increasing width of Au nanorod. On the contrary, the FWHM significantly becomes narrower as the loss of SiO_2 is considered as shown in

Fig. 2. This reveals that the Q-value enhancement is due to the phonon absorption of SiO_2 at 8-12 μm.

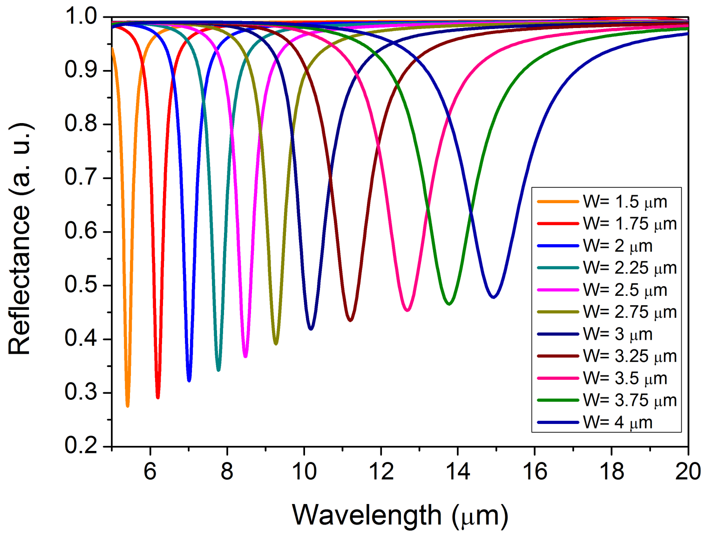

Fig. S3 Simulated reflection spectrum of Au nanorod array with lossless SiO_2 layer.

Reference:

1. F.A. Jenkins and H.E. White, *Fundamentals of Optics*, 4th ed., McGraw-Hill, Inc. , **1981.**
